# Prevalence of Vitamin B12 Deficiency Among Adult Male Outpatients in a Hospital Setting: A Retrospective Cross-Sectional Study at Hazm Mebaireek General Hospital, Qatar

**DOI:** 10.7759/cureus.99098

**Published:** 2025-12-13

**Authors:** Mohammed K Farooqi, Ammar A Abdelrahman, Amena Begum, Nabeel F Allobaney, Abdulqadir J Nashwan

**Affiliations:** 1 Department of Internal Medicine, Hamad Medical Corporation, Doha, QAT; 2 Department of Nursing, Hamad Medical Corporation, Doha, QAT; 3 Department of Nursing and Midwifery Research, Hamad Medical Corporation, Doha, QAT

**Keywords:** borderline vitamin b12 status, nutritional screening, outpatient clinics, south asian population, vitamin b12 deficiency

## Abstract

Introduction

Vitamin B12 deficiency is a common nutritional deficiency, particularly among individuals with poor dietary intake or malabsorption disorders. Given the potential for serious health consequences-including anemia, neuropathy, and cognitive decline-routine vitamin B12 monitoring is critical, particularly in patients with chronic conditions. This study aimed to determine the prevalence of vitamin B12 deficiency and borderline deficiency in male outpatients attending a hospital clinic.

Methods

A retrospective cross-sectional study was conducted, including male patients attending outpatient clinics at Hazm Mebaireek General Hospital between April 1 and October 31, 2022. Serum vitamin B12 levels were measured, and patients were categorized into three groups: deficient (<150 pmol/L), borderline deficiency (151-221 pmol/L), and adequate (≥222 pmol/L). Descriptive and statistical analyses were performed to assess the prevalence of deficiency and borderline deficiency.

Results

Among 1173 male outpatients, 13.2% had vitamin B12 deficiency, and 23.8% exhibited borderline deficiency, totaling 37.0% with suboptimal B12 status. A higher prevalence was observed among South Asian participants, suggesting dietary influences.

Conclusion

Vitamin B12 deficiency and borderline status are common among male outpatients attending medical outpatient clinics, especially among South Asian expatriates. Routine screening in high-risk groups may prevent irreversible complications and should be considered in national guidelines.

## Introduction

Vitamin insufficiency remains a significant global public health concern, with vitamin B12 (cobalamin) deficiency recognized as a distinct clinical condition for almost a century [1]. Despite this long-standing awareness, its true prevalence remains difficult to quantify due to the absence of universally accepted diagnostic thresholds and variation in clinical practice [2]. The clinical presentation of vitamin B12 deficiency is highly variable, ranging from subtle symptoms such as fatigue and mild cognitive changes to severe neuropsychiatric and hematological complications. These features are often nonspecific and therefore easily overlooked, leading to underdiagnosis and potentially irreversible morbidity when treatment is delayed [3]. Given the central role of vitamin B12 in red blood cell formation, neurological function, and DNA synthesis, timely identification and management of deficiency are essential.

The classification of vitamin B12 status continues to evolve across clinical guidelines, with some consensus emerging but notable debate remaining. Shipton et al. [1] define marginal depletion as serum cobalamin concentrations of 148-221 pmol/L, whereas values below 148 pmol/L, particularly when accompanied by clinical features or abnormal hematologic indices, are considered consistent with deficiency. In contrast, Ankar et al. [4] suggest that serum B12 levels <148 pmol/L alone are sufficient for diagnosis, irrespective of accompanying clinical manifestations. In this study, we adopted a three-tiered classification system to distinguish apparent deficiency (<150 pmol/L), a borderline "gray zone" (151-221 pmol/L), and normal status (≥222 pmol/L), acknowledging that biochemical insufficiency may precede overt clinical disease [5-11].

Notably, a substantial proportion of the population, particularly expatriate male workers from South Asian countries, may be predisposed to deficiency due to dietary habits. Vegetarian and predominantly plant-based diets are reported in up to one-third of South Asian populations, compared to significantly lower rates among Arab populations [3]. Given that natural sources of vitamin B12 are primarily animal-derived, such dietary patterns increase individuals' risk of inadequate intake. Additionally, factors such as limited dietary acculturation, malabsorption disorders, and possible autoimmune mechanisms may further contribute to deficiency in this group.

Medical outpatient clinics serve as a critical point of care for early detection, as many individuals present with one or more risk factors, including long-term metformin use, advancing age, *Helicobacter pylori* infection, or chronic gastrointestinal conditions [5]. However, early manifestations of deficiency-such as peripheral neuropathy or cognitive changes-are frequently misattributed to diabetes or age-related decline [12-14]. The country's unique demographic composition shapes the clinical context of B12 deficiency in Qatar. While previous local research has primarily focused on high-risk groups such as patients with diabetes, where deficiency rates as high as 30.7% have been reported among metformin users [15], limited data exist regarding the broader outpatient population. Furthermore, gaps in follow-up and lack of standardized screening protocols result in missed opportunities for intervention, even though timely and inexpensive supplementation can prevent severe and often irreversible neurological damage [1,4]. The clinical and economic burden associated with late diagnosis underscores the need for evidence-based screening strategies tailored to Qatar's diverse population. This study addresses a key public health priority, establishing the first population-level estimate of B12 status among male outpatients in Qatar, with emphasis on the most significant demographic subgroup (South Asian expatriates), to inform targeted screening and prevention strategies.

Therefore, this study aimed to (1) establish the prevalence of vitamin B12 deficiency and borderline deficiency among male outpatients attending a general medical clinic, (2) identify high-risk demographic subgroups, and (3) provide evidence to guide national recommendations for routine B12 screening and management. 

## Materials and methods

Study design and setting 

This study employed a retrospective cross-sectional design to analyze vitamin B12 levels among patients at Hazm Mebaireek General Hospital in Doha, Qatar. The research was conducted over six months from April 1 to October 31, 2022. This setting is particularly relevant given the hospital's diverse patient demographic, which includes a significant number of South Asian expatriate groups that may have specific dietary and health patterns impacting vitamin B12 levels.

Participants and inclusion criteria 

The study targeted male adults aged 18 years and older who visited internal medicine outpatient clinics during the specified timeframe. However, patients with incomplete medical records or lacking B12 measurement data were excluded from the study. This criterion was essential to maintaining data integrity and reliability in assessing the prevalence of vitamin B12 deficiency.

Data collection 

Data collection was meticulously executed using electronic medical records, facilitating an efficient and comprehensive review of patient information. Key data extracted included demographic details such as age and ethnicity, as well as clinical comorbidities like diabetes, hypertension, and chronic kidney disease. Laboratory results were also recorded, specifically focusing on vitamin B12 serum levels, which are critical for understanding the health status of the study population.

Laboratory methods 

Vitamin B12 levels were assessed using an electrochemiluminescence immunoassay, a recognized and validated method for measuring cobalamin status in clinical settings. The study used established international clinical thresholds to categorize participants' serum vitamin B12 concentrations [8-9]. Deficiency was defined as levels below 150 pmol/L, indicating a significant lack of vitamin B12. Levels ranging from 151-221 pmol/L were classified as borderline deficiency, representing a physiological "gray zone" where functional impairment might arise without observable hematologic abnormalities. Lastly, levels equal to or greater than 222 pmol/L were designated as usual, suggesting adequate vitamin B12 stores necessary for both hematologic and neurologic functions.

Statistical analysis 

The analysis used descriptive statistics to summarize the participants' demographic and clinical characteristics. To compare differences across the designated vitamin B12 groups, statistical tests such as ANOVA and Chi-square tests were employed. Additionally, a multivariable logistic regression analysis was conducted to identify independent predictors of vitamin B12 deficiency, accounting for various demographic and health factors. All statistical analyses were performed using SPSS version 26 (IBM Inc., Armonk, New York), with a significance threshold of p<0.05, ensuring robust, meaningful findings.

This comprehensive approach to study design, participant selection, data collection, and analysis equips the research with a solid framework for understanding the prevalence and predictors of vitamin B12 deficiency among the studied population. The insights from this research could ultimately inform clinical practices and interventions to address vitamin B12 deficiency in similar demographic groups.

## Results

A total of 1173 male outpatients were included in the analysis. The prevalence of vitamin B12 deficiency (<150 pmol/L) was 13.2%, and an additional 23.8% had borderline levels (151-221 pmol/L); thus, 37.0% of the cohort had suboptimal vitamin B12 status. The remaining 63.0% of participants had serum levels within the normal range (≥222 pmol/L). These findings are summarized in Table 1.

**Table 1:** Prevalence of vitamin B12 deficiency categories Cutoffs per WHO (2004) [9] and British Society for Haematology (2024) [8].

Category	Number of participants n (%)	95% confidence interval (%)
Deficient (<150 pmol/L)	155 (13.2%)	11.3% - 15.2%
Borderline (151–221 pmol/L)	279 (23.8%)	21.4% - 26.4%
Normal (≥222 pmol/L)	739 (63.0%)	60.1% - 65.8%
Total	1173 (100%)	-

Significant differences in serum vitamin B12 concentrations were observed across ethnic groups. South Asian participants, who comprised the majority of the sample (74.1%), had the lowest mean B12 levels (298 ± 220 pmol/L) and median values (240 pmol/L), compared with GCC/Arab (median 350 pmol/L) and African/Other groups (median 370 pmol/L). This difference was statistically significant (p<0.001), indicating a higher burden of deficiency among South Asians. Age-related variation was also observed, with older participants (>45 years) showing slightly higher median B12 levels than younger participants, though with greater variability. Detailed comparisons are provided in Table 2.

**Table 2:** Demographic and clinical variations in vitamin B12 levels SD - standard deviation; IQR - interquartile range; GCC - Gulf Cooperation Countries

Characteristic (ethnicity & age group)	n (%)	Mean ± SD (pmol/L)	Median	IQR	p-value / statistical test	Interpretation
Ethnicity						
South Asian	876 (74.1)	298 ± 220	240	180- 310	<0.001 (ANOVA, F (2,1170) =10.47)	Significantly lower B12
GCC / Arab	154 (13.1)	378 ± 210	350	300- 400	Reference	Higher B12
African / Other	143 (12.2)	375 ± 200	370	320- 420	0.82 vs GCC	Comparable to GCC
Age group (years)						
≤35 years	258 (22.0)	299 ± 220	240	180- 310	-	Lower B12, wider range
35-45 years	406 (34.6)	302 ± 211	250	190- 320	0.032 (ANOVA, F (2,1170) =3.47)	Slightly higher
>45 years	509 (43.4)	341 ± 305	280	210- 380	-	Highest mean

The multivariable logistic regression model included age (continuous), ethnicity (categorical), and all major comorbidities (diabetes, hypertension, chronic kidney disease, hypothyroidism) as covariates, based on a priori clinical relevance and literature review, not stepwise selection, to avoid data-driven bias. Multicollinearity was assessed using variance inflation factors (all VIFs <2.0). Model fit was evaluated using the Hosmer-Lemeshow test (p=0.31), indicating good calibration. Compared with South Asian participants, GCC/Arab individuals had a 35% lower odds of deficiency (AOR 0.65, 95% CI 0.45-0.92), while those categorized as African/Other had a 59% lower odds (AOR 0.41, 95% CI 0.28-0.60). Interestingly, patients with diabetes had significantly lower odds of deficiency (AOR 0.53, 95% CI 0.40-0.70), likely reflecting more frequent healthcare encounters and routine supplementation. Age, chronic kidney disease, hypertension, and hypothyroidism were not significant predictors. Full model results are presented in Table 3.

**Table 3:** Logistic regression analysis of predictors for vitamin B12 deficiency (≤150 pmol/L) Reference group for ethnicity: South Asian. OR <1 indicates reduced odds of deficiency. Model adjusted for: age, ethnicity, diabetes, hypertension, CKD, hypothyroidism. Reference group: South Asian, non-diabetic, etc. GCC - Gulf Cooperation Countries; CKD - chronic kidney disease

Predictor variable	Adjusted odds ratio (95% CI)	p-value	Interpretation
GCC/Arab (vs. South Asian)	0.65 (0.45–0.92)	0.02	Lower odds of deficiency
African/Other (vs. South Asian)	0.41 (0.28–0.60)	<0.001	Much lower odds
Diabetes (Yes vs. No)	0.53 (0.40–0.70)	<0.001	Lower odds, likely due to supplementation
Age (per year)	1.01 (0.99–1.03)	0.27	Not significant
CKD (Yes vs. No)	1.12 (0.85–1.47)	0.42	Not significant
Hypertension (Yes vs. No)	0.93 (0.71–1.22)	0.61	Not significant
Hypothyroidism (Yes vs. No)	1.08 (0.82–1.42)	0.59	Not significant

## Discussion

Vitamin B12 deficiency remains an important yet under-recognized global health issue, particularly in regions characterized by diverse cultural and dietary practices. In this cohort of 1173 male outpatients, 37% demonstrated either vitamin B12 deficiency or borderline status, a prevalence notably higher than estimates reported for general Western populations but comparable to patterns described in South Asian communities, where lower intake of animal-source foods and limited consumption of fortified products are frequently observed [16]. Given the high proportion of South Asian expatriates in this population, an important question arises as to whether this demographic distribution may, at least in part, account for the elevated prevalence observed.

This pattern is consistent with evidence from large-scale studies conducted in South Asia, where vitamin B12 deficiency has been reported at rates exceeding 50% in some settings [17]. Likewise, studies among South Asian migrants in high-income countries have documented persistently higher deficiency rates than those seen in host populations, raising questions about the potential influence of culturally shaped dietary practices and access to fortified foods [18]. In contrast, lower deficiency rates reported in Europe and North America are generally discussed in the context of higher animal-source food intake and long-standing vitamin fortification policies [16].

A notable finding in our study is the lower odds of deficiency among individuals with diabetes, despite the well-established association between metformin use and vitamin B12 depletion [19]. This pattern likely reflects Qatar's structured diabetes care practices, in which routine monitoring and preventive B12 supplementation are commonly integrated into chronic disease management [20]. This observation underscores the potential value of targeted supplementation for high-risk groups within primary care systems.

Beyond classical hematologic and neurologic complications, vitamin B12 deficiency has also been linked to cognitive impairment, depressive symptoms, and increased cardiovascular risk through elevated homocysteine levels [21]. These broader systemic effects emphasize the clinical importance of detecting and managing deficiency even in asymptomatic individuals. However, serum B12 alone may not fully capture intracellular cobalamin status. Incorporating functional biomarkers such as methylmalonic acid or holotranscobalamin could improve diagnostic specificity, particularly for individuals in the borderline range [22].

From a public health perspective, the combined prevalence of deficiency and borderline status in this cohort approaches the global prevalence of prediabetes among adults of comparable age, highlighting its relevance as a non-communicable nutritional condition [23]. Given that oral cobalamin supplementation is safe, inexpensive, and effective, targeted screening of high-risk populations, particularly South Asian expatriates, offers favorable cost-effectiveness. Economic analyses in other high-income settings suggest that early diagnosis and supplementation substantially reduce long-term healthcare expenditure associated with irreversible neurological injury [24]. At the policy level, population-level strategies such as fortifying staple foods or commonly consumed plant-based beverages with vitamin B12 could be explored, drawing from successful fortification initiatives in Canada and the United States [25].

Study limitations

This study has several limitations. It was retrospective and limited to male outpatients, reducing generalizability to women and community-based populations. Vitamin B12 status was assessed solely through serum concentrations, without complementary functional markers such as methylmalonic acid, which may lead to misclassification, especially in the borderline range. Dietary patterns and supplement use were not systematically captured, so ethnicity was used as a proxy for dietary risk. Additionally, the cross-sectional design precludes causal inference regarding clinical outcomes.

Future directions

Future research should include female populations, integrate dietary and supplement-use data, and incorporate functional biomarkers to refine diagnostic thresholds. Longitudinal studies may clarify the progression from biochemical deficiency to symptomatic disease, while interventional trials comparing oral and intramuscular supplementation across cultural groups would support evidence-based management strategies.

## Conclusions

This study highlights a silent epidemic of vitamin B12 insufficiency among adult male outpatients in Qatar, with 37% exhibiting suboptimal levels. South Asian expatriates bear the highest burden, likely due to diet and genetic factors. Given the low cost, safety, and high impact of early detection, and the risk of irreversible neurological damage, routine B12 screening should be integrated into preventive care for high-risk populations, particularly in multicultural settings like Qatar.
